# IFITM1 expression determines extracellular vesicle uptake in colorectal cancer

**DOI:** 10.1007/s00018-021-03949-w

**Published:** 2021-10-05

**Authors:** Andrea Kelemen, Idan Carmi, Ádám Oszvald, Péter Lőrincz, Gábor Petővári, Tamás Tölgyes, Kristóf Dede, Attila Bursics, Edit I. Buzás, Zoltán Wiener

**Affiliations:** 1 Department of Genetics, Cell and Immunobiology, Semmelweis University, Budapest, Hungary; 2 Department of Anatomy, Cell and Developmental Biology, Eötvös Loránd University of Sciences, Budapest, Hungary; 3 Premium Postdoctoral Research Program, Hungarian Academy of Sciences, Budapest, Hungary; 4 1st Department of Pathology and Experimental Cancer Research, Semmelweis University, Budapest, Hungary; 5 Uzsoki Hospital, Budapest, Hungary; 6 ELKH-SE Immune-Proteogenomics Extracellular Vesicle Research Group, Semmelweis University, Budapest, Hungary; 7 HCEMM-SE Extracellular Vesicle Research Group, Budapest, Hungary

**Keywords:** IFITM1, Exosome, Patient-derived organoid, CRC

## Abstract

The majority of colorectal cancer (CRC) patients carry mutations in the *APC* gene, which lead to the unregulated activation of the Wnt pathway. Extracellular vesicles (EV) are considered potential therapeutic tools. Although CRC is a genetically heterogeneous disease, the significance of the intra-tumor heterogeneity in EV uptake of CRC cells is not yet known. By using mouse and patient-derived organoids, the currently available best model of capturing cellular heterogeneity, we found that *Apc* mutation induced the expression of interferon-induced transmembrane protein 1 (Ifitm1), a membrane protein that plays a major role in cellular antiviral responses. Importantly, organoids derived from IFITM1^high^ CRC cells contained more proliferating cells and they had a markedly reduced uptake of fibroblast EVs as compared to IFITM1^low/−^ cells. In contrast, there was no difference in the intensity of EV release between CRC subpopulations with high and low IFITM1 levels. Importantly, the difference in cell proliferation between these two subpopulations disappeared in the presence of fibroblast-derived EVs, proving the functional relevance of the enhanced EV uptake by IFITM1^low^ CRC cells. Furthermore, inactivating IFITM1 resulted in an enhanced EV uptake, highlighting the importance of this molecule in establishing the cellular difference for EV effects. Collectively, we identified CRC cells with functional difference in their EV uptake ability that must be taken into consideration when using EVs as therapeutic tools for targeting cancer cells.

**Supplementary Information:**

The online version contains supplementary material available at 10.1007/s00018-021-03949-w.

## Introduction

Colorectal cancer (CRC) is a leading cause of cancer-related deaths in developed countries. In the vast majority of CRC patients, *APC* mutation is a central genetic event leading to the ligand-independent activation of the Wnt pathway and the uncontrolled proliferation of adenoma cells. CRC is both genetically and phenotypically diverse, and other driver mutations often occur such as *KRAS*, *TP53*, inactivation of the TGFβ signalling pathway, etc. This accumulation of mutations results in the progression of adenomas to invasive carcinomas. Recently, a common clustering of CRC has been suggested with CMS1-CMS4 subtypes that have characteristic mutation profiles [1]. However, CRC not only shows a large diversity among patients, but also an intra-tumoral cellular heterogeneity that is critical in therapy. Of note, one of the most state-of-the-art methods to capture this intra-tumoral heterogeneity of human cancers is the organoid technology [2, 3]. Organoids are derived from patient samples and they are cultured in 3D matrices, such as Matrigel under well-defined conditions.

Interestingly, the activity of the Wnt pathway is influenced not only by mutations but also by other factors as well. As a consequence, Wnt signalling intensity is different among cells from the same tumor [4]. This leads to specific Wnt target gene expression patterns and tumor cells with functional diversity. For example, the Wnt target LGR5 is induced only at a high Wnt activity. Although nowadays the identity and the function of CRC stem cells are intensively discussed, LGR5 expression is agreed to mark a CRC cell population with stem cell properties [5, 6].

The accumulation and activation of fibroblasts are hallmarks of some CRC subtypes. Importantly, there is a strong negative correlation between the amount of cancer-associated fibroblasts (CAF) and the time to disease relapse in CRC [7]. Furthermore, stromal gene expression signature predicts poor survival in patients [7]. CRC cells may undergo epithelial-mesenchymal transition and they may acquire a stromal expression pattern for a limited set of genes [8]. However, the predictive value of the stromal signature can at least partially be attributed to CAFs in the tumor tissues. Importantly, by secreting growth factors, CAFs contribute to the intra-tumoral cellular heterogeneity as well [4].

Extracellular vesicles (EV) are membrane-enclosed structures carrying biologically important molecules, such as proteins, lipids and RNAs between cells. Thus, they represent a special form of intercellular communication. EVs can be classified according to their biogenesis, and they can be characterized by their biophysical or biochemical properties, too [9]. While exosomes are derived from the multivesicular bodies of the endosomal-lysosomal compartment, microvesicles are shed from the plasma membrane and they represent a larger EV population. In addition, large vesicles e.g. apoptotic bodies released by dying cells are regarded as EVs as well [10]. Since isolating EVs based on their cellular origin is difficult, they are often characterized by their size upon purification with differential centrifugation and ultracentrifugation [11]. Since EVs can be edited and loaded with specific molecules which are then transported in a protected way, furthermore, EVs are efficiently taken up by target cells, they hold a great promise for cancer therapies as well [12]. However, molecules important in EV uptake and whether specific CRC subpopulations can be targeted by EVs more efficiently than others, are not yet known.

Members of the interferon-induced transmembrane (IFITM) family regulate virus uptake in different compartments of the cells, thus, they critically contribute to cellular resistance against a wide range of membrane-surrounded viruses [13]. Among IFITM proteins, IFITM1 functions in the plasma membrane [14, 15]. By using mouse intestinal and patient-derived organoids, here we provide evidence that differential IFITM1 expression marks CRC cells with a significantly different capacity for EV uptake. Importantly, IFITM1 functions not only as a marker of these cell populations, but also critically inhibits EV uptake. In addition, our results show that intra-tumoral cellular heterogeneity of CRC in IFITM1 expression has a major impact on fibroblast-derived EV function. Since the potential use of EVs in therapies is critically influenced by their uptake in tumor cells, our results may have an important consequence when designing EV-based targeting tools.

## Materials and methods

### Cell culture

HT29 cells (ATCC HTB-38, American Type Culture Collection) and normal human colon fibroblasts (CCD-18Co, ATCC-1459) were cultured in DMEM containing 4500 g/L glucose (Gibco), 10% FBS (Biosera), glutamine (Sigma) and 1× penicillin/streptomycin (Gibco). Cells were washed with phosphate buffered saline (PBS) three times and cultured in serum-free medium for 2 days. The medium was then changed and EVs were collected for 2 days. Cell number was counted in a Burker chamber. We only used cells with low (< p9) passage numbers after receiving them from ATCC. Cell cultures were regularly tested for Mycoplasma contamination with Hoechst staining and they were negative in our studies.

### Human adenoma and CRC organoid cultures

The Medical Research Council of Hungary (ETT-TUKEB, No 51323-4/2015/EKU) approved the experiments with human samples and informed consent was obtained from the patients. CRC and adenoma patient-derived samples were processed according to previously published protocols [16]. Digested cell clusters were isolated by centrifugation at 650*g* for 2 min and they were embedded into growth factor-reduced, phenol red-free Matrigel (Corning). In addition, we used the CRC organoid lines previously established in our research group [17]. Organoids were cultured in CRC medium containing basal medium (advanced DMEM/F12, 10 mM HEPES (Sigma), penicillin/streptomycin, glutamine) and additionally B27 supplements (Gibco), 1 mM N-Acetyl-Cysteine, 10 mM Nicotinamide, 50 ng/mL EGF (Peprotech), 500 nM A83-01 (Sigma), 10 μM SB202190-Monohydrochloride (Sigma). Furthermore, Y27632 (Rho-kinase inhibitor, Sigma) was added for 3 days after passaging to avoid anoikis. Organoids were removed from Matrigel every 5–6 days mechanically, they were centrifuged at 650*g* for 5 min and then digested with TrypLE (Thermo Fisher) for 5–10 min. CRC cell clusters were then washed with basal medium and embedded into Matrigel again in a 1:3 ratio. Clinical data of the patients and characterization of the organoids are in Table S1. Organoids #1–3 have already been published [17].

### Normal human colon organoids

Normal colon tissue samples were collected from patients undergoing CRC surgical operation with a distance of more than 3 cm from the tumors [16]. Human colonic crypts were isolated according to [16] with some modifications. Samples were cut into < 0.5 cm pieces, washed with PBS five times, and incubated in PBS containing 2 mM EDTA (Sigma) for 30 min at 4 °C. The tubes had been pre-coated with 0.1% bovine serum albumin (BSA, Sigma) before the isolation procedure. Fractions were taken with PBS + 0.1% BSA into tubes containing 3 mL advanced DMEM/F12 medium (Gibco). Fractions with crypts were embedded into Matrigel and cultured in human organoid medium composed of CRC medium, supplemented with 100 ng/mL human noggin (Peprotech), 1000 ng/mL human R-Spondin1 (R&D Systems), 100 ng/mL murine Wnt3a (Peprotech) and 1 nM gastrin (Sigma). The medium was changed every 3 days.

### Mouse intestinal organoid cultures

The veterinary authority (Pest County Government Office, Hungary) approved the experiments with mice. Small intestinal crypts from C57Bl/6J (Jackson Laboratory) were isolated according to previously published protocols [18]. Approximately 1000 crypts were embedded into Matrigel (Corning, 20 μL/well in 48-well plates) and cultured in small intestinal medium (SIM) contained advanced DMEM/F12 with N2 and B27 supplements (Gibco), 10 mM HEPES (Sigma), 1 µM N-Acetyl-Cysteine (Sigma), glutamine, penicillin/streptomycin, antibiotic/antimycotic mix (Gibco), 100 ng/mL noggin and 50 ng/mL EGF (Peprotech), 500 ng/mL mouse R-Spondin1 (R&D Systems, Bio-Techne). Organoids were removed from Matrigel every 5–6 days, mechanically disrupted by pipetting, they were centrifuged at 650*g* for 5 min and then embedded into Matrigel.

To produce *Apc*-mutant organoids, we used a previously published sgRNA sequence (sgRNA4, [19]) and cloned it into the lentiCRISPR v2 plasmid (Addgene 52961). *Apc*-mutant organoids were produced according to [17] and [19] and they were selected by omitting R-Spondin1, EGF and noggin from the culture medium 3 days after transfection. Selected *Apc*-mutant organoids were used > 6 days after removing the growth factors.

### EV isolation for functional experiments

Serum-free medium from cultured cells was collected after 2 days. In some experiments, fibroblasts or HT29 cells were cultured with Vybrant™ Cell-Labeling Solution DiI dye for 10 min (Thermo Fisher, according to the Cell in Suspension protocol of the manufacturer), the cells were extensively washed with DMEM-Glutamax medium (Gibco) twice to remove the free dye. After the medium was changed to serum-free after 2 days and labelled EVs were isolated after 3 days, the supernatants were serially centrifuged at 300*g* for 5 min and 2000*g* for 20 min at 16 °C to remove cells and cell fragments. Samples were then centrifuged at 12,500*g* for 30 min. The pellet was washed with PBS, centrifuged again and used as the medium EV fraction (mEV, 12.5 K fraction). Small EVs (sEV, 100 K fraction) were ultracentrifuged (UC) at 100,000*g* for 70 min at 4 °C, the EV-containing pellet was resuspended in PBS and UC again. The EV-containing pellets were then resuspended in PBS and they were used for functional experiments.

### 2D experiments with organoid cells

2D experiments with organoid cells were carried out according to [20] with modifications. Briefly, 24-well plates (Eppendorf) or 8-well culture slides (Falcon) were pre-coated with Matrigel and basal medium mixture in case of human CRC organoids 1:20 ratio, in case of mouse organoids 1:10 ratio for 1 h at 37 °C. The liquid was then discarded and plates were dried at room temperature. 10^4^ sorted CRC cells were plated into each well. The supernatant was changed to fresh medium after 1 day and the cells were treated with DiI labelled medium-sized EVs. The treated cells were fixed with 4% paraformaldehyde (PFA) for 20 min the next day. Cells were then incubated with Phalloidin-iFluor 488 Reagent (Abcam) for 20 min. After covering samples with ProLong Diamond antifade mountant containing DAPI (Thermo Fisher), images were taken with a Zeiss LSM800 or Leica TCS SP8 confocal microscopes. For mouse intestinal organoid 2D experiments, organoids were removed from Matrigel, they were centrifuged at 650*g* for 5 min and digested with TrypLE (Gibco) until we received a single cell suspension (5–15 min). Cells were then washed with PBS and they were cultured in SIM medium with the Rho kinase inhibitor Y27632 in 24-well plates (Eppendorf). Cells were used for EV uptake experiments at 24 h.

### Nanoparticle tracking analysis (NTA)

Cells and organoids were cultured in FBS-free medium or in the chemically defined CRC medium, respectively. The supernatants were harvested after 2 days, they were serially centrifuged at 300*g* for 5 min and at 2000*g* for 20 min. After centrifugation, 100 µL supernatant was diluted to 1 mL in PBS and particle concentrations and size distribution were measured on a ZetaView Z-NTA instrument (Particle Metrix). For each measurement, the cell positions were scanned at 25 °C. The following camera settings were used: auto expose, gain: 28.8, offset: 0, shutter: 100, sensitivity: 80. The videos were analyzed with a minimum area of 5, a maximum area of 1000, and minimum brightness of 20 by the ZetaView Analyze software 8.05.10. When the EV release from different experimental groups was compared, cells were cultured under the same conditions (medium volume, time, tissue culture dish format) and particle concentration data were then normalized to cell number.

### Functional studies with EVs

10^8^ small EVs in 10 µL were added to each well with CRC organoids (200 µL medium, 20 µL Matrigel, 48-well tissue culture plates, Eppendorf). In some experiments, mEVs were incubated with the organoids in Eppendorf tubes in a total volume of 10 µL for 1 h before embedding them into the 3D matrix. DiI labelled mEVs or labelled sEVs were added to 2D cell cultures (10^6^ cells in 500 µL medium) and the EV uptake was analyzed by confocal microscopy or flow cytometry (see below). The pixel intensity distribution on confocal images was calculated using a custom Python script (available from the authors upon request). Images were segmented using the Cellpose algorithm [21] to determine the cell boundaries for each cell. Pixel values were then extracted within the cell boundaries from the red channel to characterize EV uptake intensity.

### Liposome production

The production and characterization of liposomes have been previously described [17]. The liposomes had a mean diameter of 105 nm. They were labelled with 2.5 µL DiO fluorescent dye (Thermo Fisher) in 500 µL PBS for 10 min at 37 °C. They were then ultracentrifuged at 100,000*g* for 70 min at 4 °C, washed with PBS and ultracentrifuged again. Cells were treated with 10^8^ liposomes in 10 µL PBS.

### Flow cytometry and cell sorting

Organoids were removed from the 3D matrix, they were centrifuged at 650*g* for 5 min, washed with PBS, mechanically disrupted by vigorous pipetting and they were then digested with TrypLE (Gibco, Thermo Fisher) until the organoids were dissociated into single cells (5–10 min). They were taken up in FACS buffer (PBS, 1 mM EDTA, 25 mM HEPES, 1% BSA). Cells were labelled with primary antibodies for 15 min and then with secondary antibodies for 15 min at room temperature. 10,000 events were measured with a FACSCalibur instrument (Beckton Dickinson) or cell subpopulations were sorted by a fluorescent cell sorter (Sony SH800S) into tubes. Sorted cells were then centrifuged at 650*g* for 10 min at 4 °C and 10,000–20,000 cells were embedded into 20 µL Matrigel droplets. The same cell numbers were used among experimental groups within the same experiment.

### Inactivating IFITM1

Single cells produced from CRC organoids (see above) were suspended at 2 × 10^5^ cells/mL in 16.4 µL Nucleofector Solution and 3.6 µl Supplement (Amaxa SF Cell Line 4D-Nucleofector X Kit S, Lonza) in the presence of 1 µg IFITM1 CRISPR/Cas9 KO and 1 µg IFITM1 CRISPR/Cas9 HDR plasmids (Santa-Cruz Biotechnology, sc-416878 and sc-416878-HDR). Cells were electroporated with a 4D-Nucleofector (Lonza) instrument using the DN 100 program. Cells were then embedded in Matrigel in the presence of CRC medium supplemented with Y27632. Successfully targeted cells were selected with 4 µg/mL puromycin (Merck) starting on day 4 of culturing. Organoid cells > 14 days after nucleofection were used in our experiments.

### Semi-quantitative analysis of EVs by anti-CD63 or anti-CD81-coated beads

Conditioned media from fibroblasts or CRC organoids were harvested after 2 days and they were centrifuged at 300*g* for 5 min and 2000*g* for 20 min. EVs were then bound to beads coated with anti-CD63 (Thermo Fisher, 10606D) or anti-CD81 (Thermo Fisher, 10616D). Before incubating with the EV-containing samples, beads had been blocked with 0.1% BSA (Sigma) for 20 min. Six µL and 20 µL of the anti-CD81 or anti-CD63-coated beads were added to 200 µL supernatant, respectively. Beads were incubated overnight at 4 °C, they were then washed with PBS three times and the EVs were labelled with FITC-anti-CD81 or PE-anti-CD63. The percentage of positive beads was detected by flow cytometry (FACSCalibur) and they were normalized to cell number.

### Immunocytochemistry

Cells were fixed in 4% PFA for 20 min, blocked, and permeabilized in blocking buffer (PBS with 0.1% BSA, 5% FBS, and 0.1% Triton X-100). Cells were then incubated with primary antibodies at 4 °C overnight and then in secondary antibodies in blocking buffer for 2 h at room temperature. After covering samples with ProLong Diamond antifade mountant containing DAPI (Thermo Fisher), images were taken with a Zeiss LSM800 or Leica TCS SP8 confocal microscope. In some experiments with the labelled medium-sized EV fraction, the blocking buffer contained 0.05% saponin (BD Biosciences) instead of Triton X-100. The antibodies used are listed in Table S2.

### Whole-mount immunostaining

CRC organoids were cultured in 8-well chamber slides (BD Biosciences), fixed in 4% PFA for 20 min and washed with PBS. Blocking and permeabilization were carried out in whole-mount blocking buffer (WBB, containing 5% FBS, 0.2% BSA, 0.3% Triton X-100 in PBS) for 30 min. Samples were incubated with primary antibodies at 4 °C overnight in WBB. After washing in PBS, labelled secondary antibodies were added overnight at 4 °C. The organoids were then mounted with ProLong Diamond antifade mountant containing DAPI (Thermo Fisher) and analyzed with Zeiss LSM800 or Leica TCS SP8 confocal microscopes. Images were evaluated by the ImageJ software. The antibodies used are listed in Table S2.

### Protein concentration measurement and simple Western (WES) analysis

Cells were cultured in serum-free medium, EVs were pelleted with serial centrifugation/ultracentrifugation, they were washed with PBS and then resuspended in 19 µL PBS and 1 µL cOmplete™ Protease Inhibitor Cocktail (Roche). For WES analysis from cells, they were taken up in 10 µl RIPA lysis buffer. The protein concentrations of the supernatants were measured with the Micro BCA Protein Assay Kit (Thermo Scientific) and NanoDrop ND-1000 spectrophotometer (Thermo Scientific). Three µL of the lysates containing 0.5 µg protein were applied to Simple Western analysis WES (ProteinSimple) following the manufacturer’s instructions. SM-W004 (for analysis between 12 and 230 kDa), DM-TP01 total protein detection kit, DM-001 anti-rabbit detection kit, DM-002 anti-mouse detection kit, and PS-ST02EZ-8 EZ Standard Pack 2 were used (ProteinSimple). The primary antibodies are listed in Table S2. The results were evaluated with the Compass for SW4.0.1 software (ProteinSimple).

### Transmission electron microscopy

The EV-containing pellet after UC was washed with PBS and then resuspended in 10 µL PBS. 5 µL droplet was dried on a 300 mesh grid (Electron Microscopy Sciences, USA). EVs were fixed with 4% glutaraldehyde for 10 min and the grid was washed with water three times. Samples were treated with 2% phosphotungstic acid, they were dried at room temperature (RT) and imaged with a JEM-1011 transmission electron microscope (JEOL, Japan) equipped with a Morada digital camera (Olympus, Japan) using the iTEM software (Olympus, Japan).

### RNA isolation and mRNA measurements from cells

Total RNA was isolated with the miRNEasy Micro Kit (Qiagen) according to the manufacturer’s protocol in 15 µL water. In some experiments, cells were directly fluorescence sorted into Qiazol (Qiagen). RNA concentration was determined with a NanoDrop instrument. Half µg RNA (in 20 µL final volume) was reverse transcribed with the SensiFAST cDNA Synthesis Kit (Bioline) and quantitative PCR reactions were carried out using the SYBRGreen method with the SensiFAST SYBR Hi-ROX Kit (Bioline) on an ABI 7900HT Fast real-time PCR instrument (384-well format, 5 µL/well volume). Results were evaluated with the following formula: relative expression level = 2^−ΔCt^, where ΔCt = Ct(gene of interest) – Ct(housekeeping gene). The primers are in Table S3.

### Bioinformatical analysis

Data in the expressional data sets GSE37926 and GSE83513 were analyzed by the GEO2R online tool. Statistical significance for determining expression difference between two groups for individual genes was determined by the default moderated t-statistics and Benjamini and Hochberg false discovery rate with a p < 0.05 threshold. HPA004810 quantity data of the Protein Atlas database (http://www.proteinatlas.org), derived from the tumor cells of the samples, were analyzed for IFITM1 immunostaining in CRC samples.

### Statistical analysis

Student’s paired or unpaired t-tests, ANOVA, Mann–Whitney U-test or Kruskal–Wallis with Dunn post hoc test were applied with *p < 0.05, **p < 0.01 and ***p < 0.005 significance levels. Microsoft Excel, and GraphPad softwares were used for statistical evaluation. Mean + SD values are shown with n = 3–5 biological replicates unless otherwise indicated.

## Results

### Ifitm1 expression is increased after Apc mutation

**Fig. 1:**
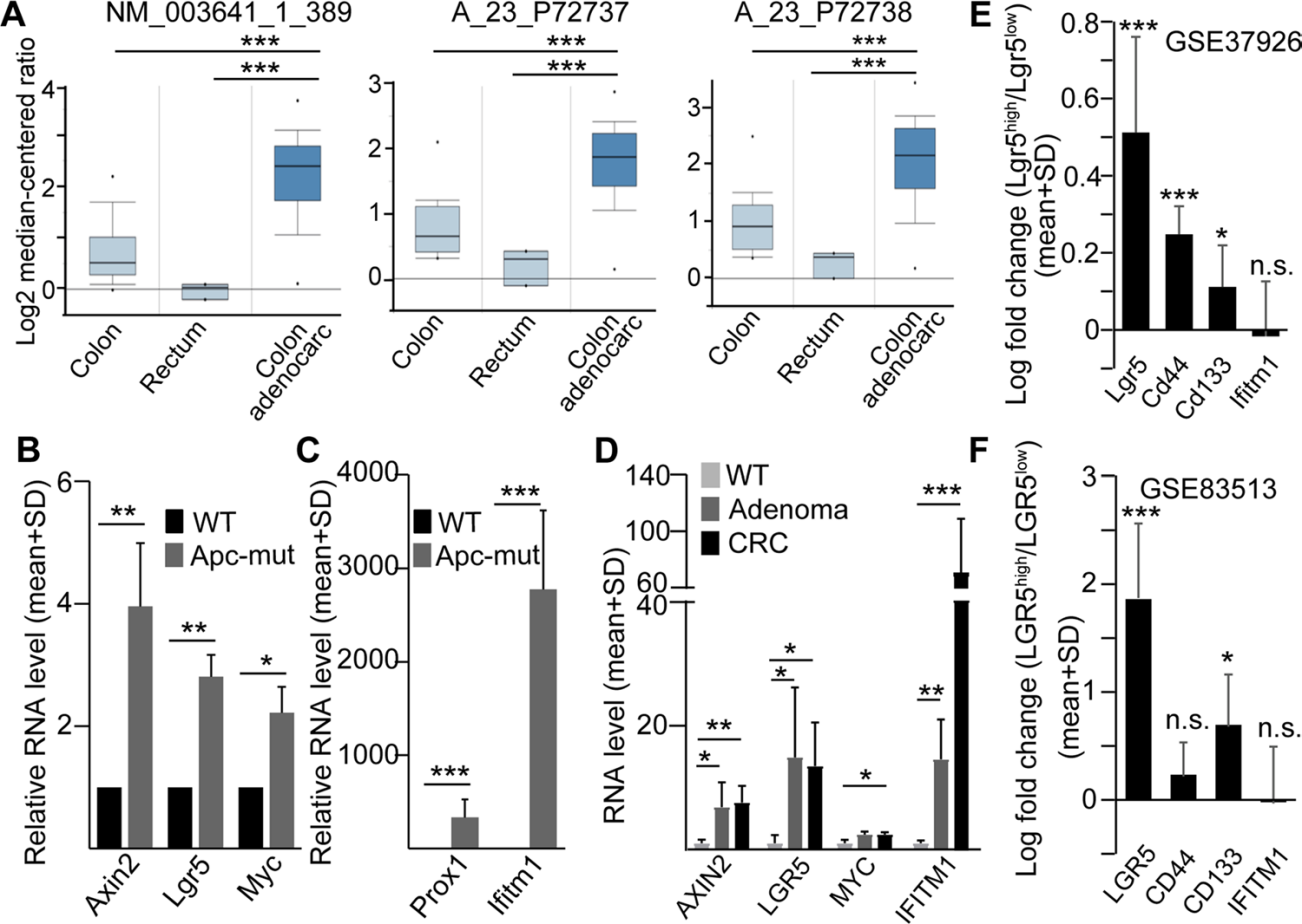
*Apc* mutation induces *Ifitm1* expression in intestinal organoids. **A** Expression analysis of the TCGA dataset in the Oncomine database (http://www.oncomine.org) for the indicated probes. **B** Relative RNA levels of the indicated genes in wild type (WT) and *Apc* mutant mouse small intestinal organoids (RT-qPCR, n = 4). **C** Relative RNA levels of *Ifitm1* and the Wnt target gene *Prox1* in the indicated mouse small intestinal organoids (RT-qPCR, n = 4). **D** *AXIN2, LGR5, MYC, IFITM1* RNA levels in organoids derived from wild type (WT), adenoma and CRC patients (RT-qPCR, n = 2 for adenoma, n = 3 for normal and CRC samples). **E** Difference in the expression levels between Lgr5^high^ and Lgr5^low^ mouse adenoma cells (bioinformatical analysis of the GSE83513 dataset with the GEO2R online tool). **F** Comparing the expression levels of *LGR5, CD44*, *CD133* and *IFITM1* between LGR5^high^ and LGR5^low^ cells in CRC patient-derived organoids (GSE83513 dataset). Unpaired t-test (**A**, **D**), paired t-test (**B**, **C**) or t-test with Benjamini and Hochberg false discovery rate (**E**, **F**) were used with *p < 0.05, **p < 0.01, ***p < 0.005, n.s.: p > 0.05

Previously we identified a set of genes that is activated by the loss of p53 and the activation of the Wnt pathway (the p53-suppressed invasiveness signature, PSIS) and which may account for the induction of invasiveness [22]. To find genes that may be involved in the malignant behavior of CRC cells and in EV uptake by CRC cells, we focused on *IFITM1,* a member of the PSIS set and the interferon-induced gene family with cell surface localization. Oncomine data analysis (TCGA colorectal data sets, http://www.oncomine.org) showed that IFITM1 is highly overexpressed in CRC samples compared to normal colon and rectum (Fig. 1A), suggesting that IFITM1 is regulated by the Wnt pathway. To test this hypothesis, we established organoid cultures from wild type (WT) mouse small intestine and introduced *Apc*-mutation to constantly activate the Wnt pathway. As expected, we observed highly elevated RNA levels of the known Wnt targets *Lgr5*, *Axin2* and *Myc* (Fig. 1B). In addition, *Apc* mutation resulted in a pronounced increase in the expression of *Prox1*, an intestine-specific Wnt target gene [23] and we detected a more than 2,500-fold increase in the expression of *Ifitm1* (Fig. 1C).

To further study the regulation of IFITM1, we isolated organoids from the normal colon (NCO) and from the tumor (CRCO) of CRC patients. In addition, we obtained samples from patients diagnosed with colon adenoma (AO) (Table S1). AOs and CRCOs were cultured without the Wnt-agonist R-Spondin1 and Wnt3a, thus, samples were selected for organoids carrying *APC* mutation and harbouring a constitutively active Wnt pathway. Interestingly, we detected higher RNA levels of not only *AXIN2*, *LGR5* and *MYC*, but also of *IFITM1* in both AOs and CRCOs as compared to NCOs (Fig. 1D).

LGR5 marks a stem cell population in CRC and in intestinal adenomas [5, 6, 24]. To decide whether Ifitm1 expression is specific for the Lgr5+ cells, we analyzed microarray data from sorted Lgr5^high^ and Lgr5^low^ mouse intestinal adenoma cells [24]. Whereas the RNA levels of *Cd44* and *Cd133* (two other genes often considered as CRC stem cell markers) were higher in the Lgr5^high^ cell population, we found no difference in *Ifitm1* (Fig. 1E). Furthermore, whereas the RNA level of *CD133* was higher in LGR5^high^ compared to LGR5^low^ human CRC organoid cells in RNA expression datasets [6], we found no difference in *IFITM1* expression between the two cell populations (Fig. 1F). Collectively, these data suggest that IFITM1 is under the regulation of the Wnt pathway, but it is not specific for the LGR5^high^ CRC cells with stem cell features.

### CRC patient-derived organoids have a heterogeneous expression of IFITM1

**Fig. 2:**
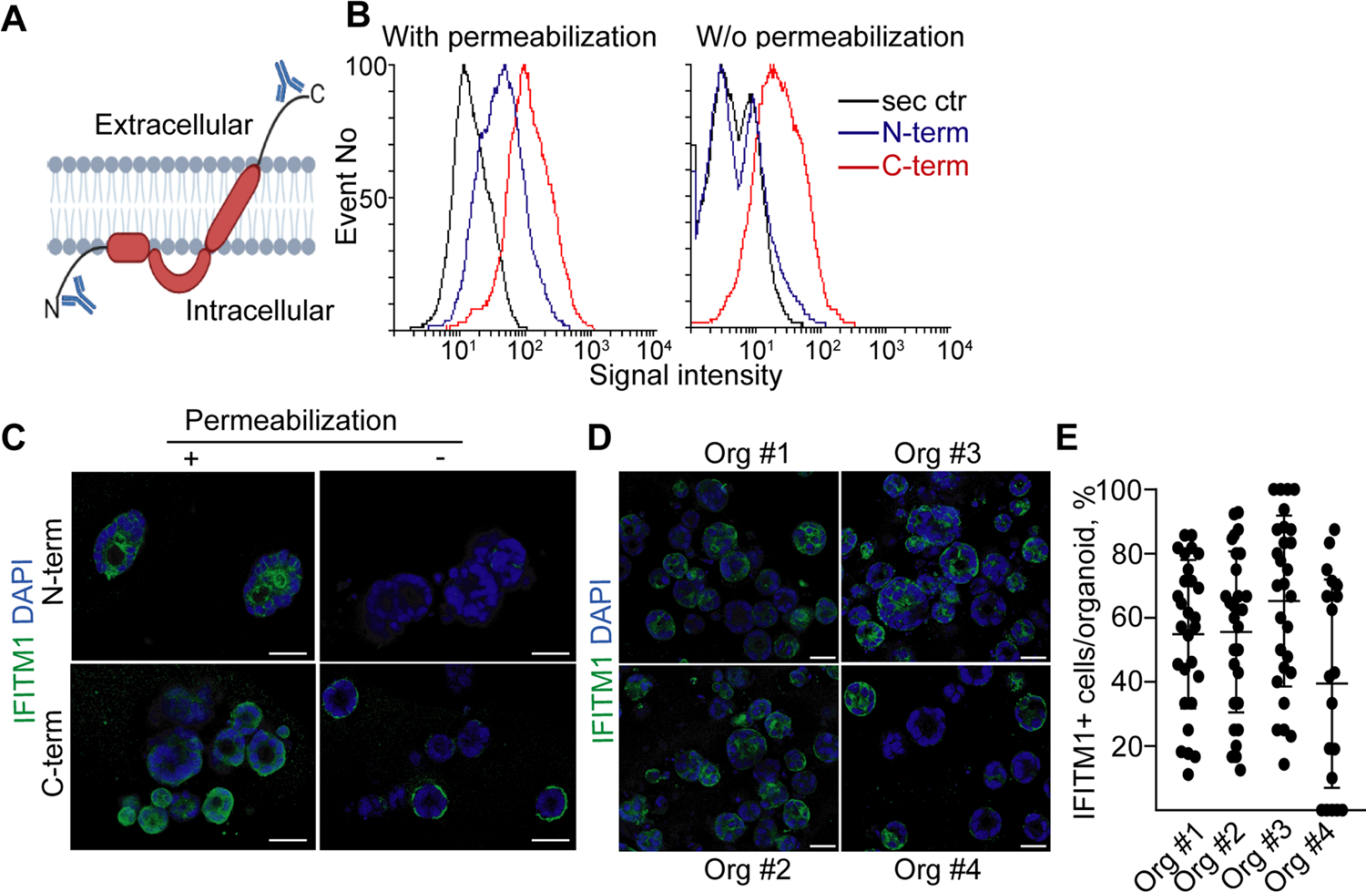
Expression level of IFITM1 in human CRC organoids. **A** The topology of IFITM1 and binding sites of the N-terminal and C-terminal antibodies. **B** Flow cytometry with antibodies specific for the N-terminal (N-term) or C-terminal (C-term) parts of IFITM1. Note that samples were either directly labelled or were fixed and permeabilized before labelling. **C** Whole-mount immunocytochemistry of CRC organoids for IFITM1 with antibodies binding to the N-terminal or C-terminal parts of the protein. Immunostaining was carried out with/without permeabilization (org #1). **D**, **E** IFITM1 expression in four CRC organoid lines with the antibody recognizing the C-terminal part of the protein. Representative images (**D**) and the quantification of confocal images (**E**) (whole-mount immunostaining). Scale bars: 50 µm (**C**, **D**)

Although the membrane topology of IFITM1 has been unknown for a long time, recent publications suggested that the C-terminal part of the protein faces the extracellular space [14, 15]. In line with these studies, we detected a flow cytometric signal with an antibody produced against the N-terminal part of IFITM1 only if cells had been permeabilized before labelling (Fig. 2A–C). In contrast, the antibody against the C-terminal part labelled cells without permeabilization as well (Fig. 2A–C). Interestingly, immunostaining showed the cellular heterogeneity in IFITM1 expression among organoids from the same patient (Fig. 2D, E). Furthermore, analysis of Protein Atlas data (http://www.proteinatlas.org) indicated that tumor cells of the same sample are heterogenous for IFITM1 level (Fig. S1a). Thus, IFITM1 expression is heterogeneous among CRC cells within organoid lines.

### The IFITM1^high^ CRC population contains more proliferating cells

**Fig. 3:**
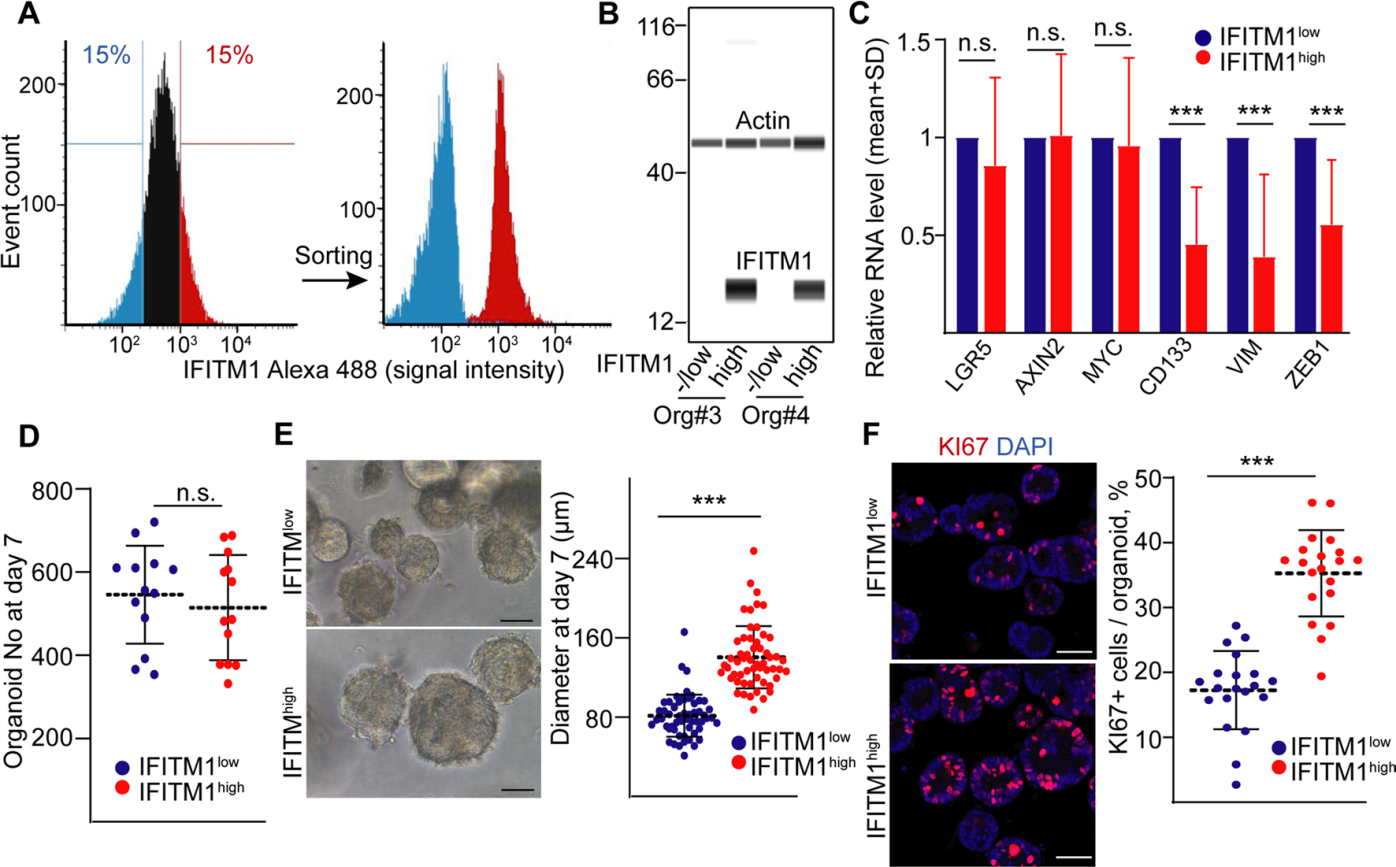
The IFITM1^high^ CRC cell-derived organoids contain more proliferating cells than IFITM1^low^ organoids. **A** The fluorescence sorting strategy for IFITM1^high^ and IFITM1^low^ cells and their analysis after sorting with flow cytometry. **B** IFITM1 protein levels of cells sorted from two CRC organoid lines (capillary-based immunoblotting). Note that actin was used as housekeeping control. **C** Relative RNA levels of the indicated genes in sorted cells (RT-qPCR, n = 4). **D** The number of organoids initiated by 20,000 sorted cells on day 7. Each dot represents an individual sorting experiment. **E** Representative images (left panel) and the diameter of CRC organoids (right panel) derived from IFITM1^low^ or IFITM1^high^ sorted cells. **F** The percentage of KI67+ proliferating cells. Representative images and their quantification from confocal images (whole-mount immunostaining). For **E** and **F**, quantifications were carried out from ten images taken from four sorting experiments. Scale bars: 50 µm (**E**, **F**). Paired t-test (**C**) and Mann–Whitney U-test (**D**–**F**) were used. ***p < 0.005, n.s.: p > 0.05

We next sorted IFITM1^high^ and IFITM1^low^ CRC organoid cells (Fig. 3A), checked the difference in the IFITM1 protein expression of the fluorescence sorted cells by flow cytometry and capillary-based immunoblotting (Fig. 3A, B) and subjected the samples to RNA analysis. Interestingly, whereas we found no difference in the RNAs of the Wnt target genes *AXIN2*, *LGR5* or *MYC*, IFITM1^high^ cells expressed the mesenchymal marker genes *VIM* and *ZEB1* at a lower level (Fig. 3C). Furthermore, we observed a decreased RNA expression of *CD133*, a gene that had been connected to stem-like cells in previous publications (Fig. 3C). Surprisingly, we detected no difference in the number of organoids initiated by the two sorted cell populations (Fig. 3D). However, the diameter of the organoids derived from IFITM1^high^ cells was significantly higher. They contained more KI67+ proliferating cells, but there was no difference in the percentage of active caspase3+ apoptotic cells (Figs. 3E, F and S1b). Parallel with these findings, we observed lower RNA levels of the differentiation markers *MUC2* (Goblet cells) and *ALPI* (enterocytes) in IFITM1^high^ cell-derived organoids (Fig. S1c), showing the shift between proliferating and other cell types. Although classical stem-like cell markers are not highly expressed in IFITM1^high^ cells, they produced organoids with a higher proliferation potential compared to the IFITM1^low^ population.

### IFITM1^high^ and IFITM1^low^ CRC cells do not differ in their intensity of EV release

**Fig. 4:**
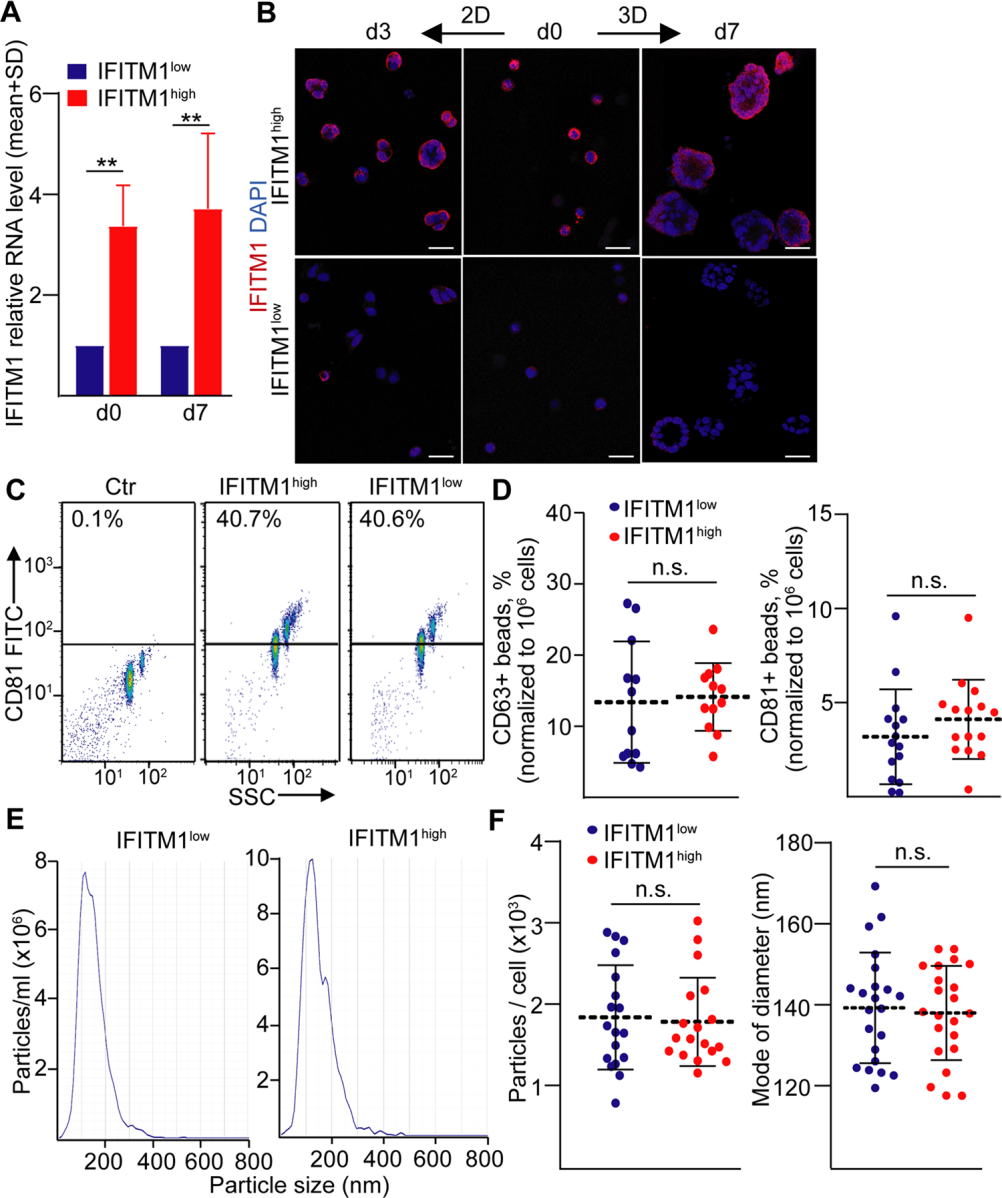
The IFITM1^high^ and IFITM1^low^ CRC cell-derived organoids do not differ in their EV release. **A** Relative *IFITM1* RNA level of sorted cells (d0) and organoids derived from the sorted cells (d7). The RNA levels (normalized to housekeeping) of IFITM1^low^ samples were taken as 1 in each experiment (RT-qPCR, n = 4). **B** Immunostaining for IFITM1 directly after sorting or after culturing in 2D or 3D conditions for the indicated time periods (immunostaining and whole-mount immunostaining, confocal microscope images). **C**, **D** The percentage of anti-CD63 or anti-CD81-coated positive beads after incubating them in cell-free medium (Ctr), in IFITM1^low^ or IFITM1^high^ organoid-derived supernatants and detected by anti-CD63 or anti-CD81 antibody, respectively (flow cytometry). Representative plots (**C**) and quantification of the data (**D**). Note that data were normalized to cell numbers. **E**, **F** Nanoparticle Tracking Analysis (NTA) of the organoid supernatants after ultracentrifugation. Representative NTA images (**E**) and data normalized to cell number (**F**). Scale bars: 50 µm (**B**). Paired t-test (**A**) and Mann–Whitney U tests (**D**, **F**) were used with **p < 0.01 and n.s.: p > 0.05

The IFITM1 cell surface protein is involved in inhibiting the uptake of some membrane-surrounded virus particles [25, 26], raising the possibility that this molecule regulates EV traffic as well. To study this question, we first determined the stability of IFITM1 expression in sorted cell populations. Organoid-derived CRC cells were viable on Matrigel-coated plates (Fig. S1d) and we found no difference in cell surface IFITM1 expression between cells cultured either 2D or 3D (Fig. S1e). Importantly, sorted cells maintained their IFITM1^high^ or IFITM1^low^ expression patterns at day 7, determined by RT-qPCR (Fig. 4A). Furthermore, IFITM1^high^ CRC cells showed a higher IFITM1 expression by immunocytochemistry even after 3 or 7 days in 2D and 3D cultures, respectively (Fig. 4B). Thus, the pattern of IFITM1 expression is maintained both in short-term 2D and 3D cultures.

Previously, we proved that small EVs (sEV, with a diameter of 50–100 nm) are preferentially released from 3D cultures into the medium [17]. sEVs can be captured from the organoid supernatants by anti-CD63 or anti-CD81-coated beads that are two widely used sEV markers and the percentage of positive beads can be detected by flow cytometry [17, 27]. We proved the presence of sEVs in CRC organoid-derived supernatants by antibody-coated beads, Nanoparticle tracking analysis (NTA), transmission electron microscopy and capillary-based immunoblot after ultracentrifugation (Fig. S2a–d). Importantly, calnexin that is not associated with sEVs [9], was detected only in cell lysates, but not in sEV preparations. In contrast, the sEV marker TSG101 was present in the ultracentrifuged pellet (Fig. S2c). We next compared the sEV release from IFITM1^high^ and IFITM1^low^ cell-derived organoids. Unexpectedly, we found no difference in the sEV concentration between the two sorted cell population-derived organoids either with the bead-based semi-quantitative method (Fig. 4C, D) or with NTA (Fig. 4E, F). We obtained similar data when sorted cells were cultured in another matrix, collagen I or in a mixture of collagen I and Matrigel (Fig. S3a–d). These results suggest that IFITM1^high^ and IFITM1^low^ CRC cells do not have an altered release of sEVs.

### IFITM1^high^ CRC cells take up less EVs

Next we studied whether cell populations with different Ifitm1 expressions have different EV uptake abilities. To address this question, we cultured cells from WT and *Apc* mutant mouse intestinal organoids in 2D conditions for short term and we added EVs isolated by centrifugation at 12,500*g* (medium EV fraction, mEV) from fibroblasts that had been pre-treated with a membrane labelling dye (Fig. S4a). Importantly, we found no increase in the percentage of active caspase+ cells after labelling, showing that this treatment does not induce apoptosis in fibroblasts (Fig. S4b). Since EVs were not directly labelled and cells were washed after applying the dye, this precluded the possibility that we measured dye aggregates. Importantly, we detected EVs in fibroblast culture supernatant by NTA measurements when isolating mEVs or sEVs (Fig. S4c). Furthermore, transmission electron microscopy proved the identity of EVs in samples after ultracentrifugation (Fig. S4d). In addition, capillary based immunoblot showed the presence of the sEV marker TSG101 only in sEV preparations, whereas we could detect calnexin in the mEV fraction (Fig. S4e) [28].

**Fig. 5:**
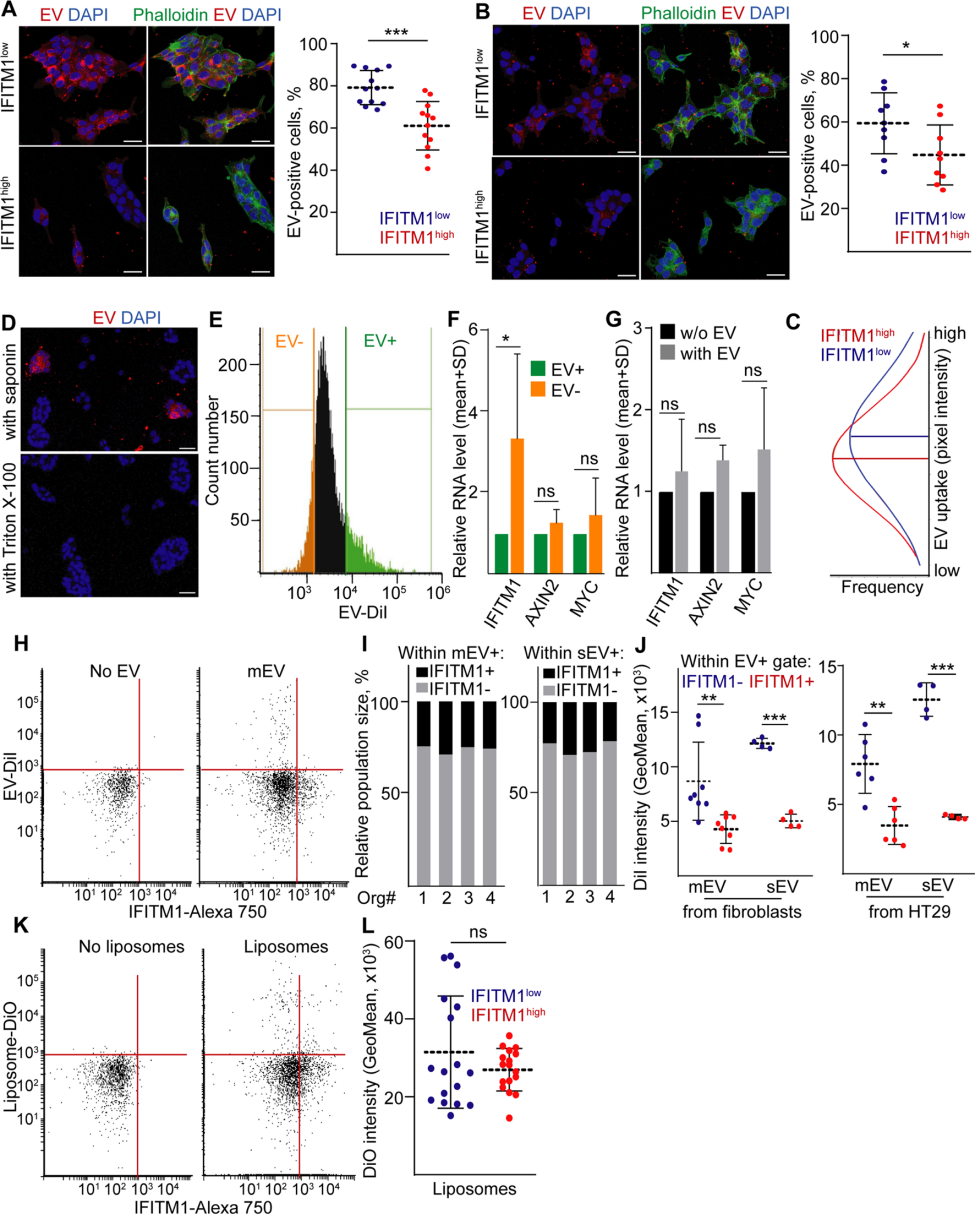
IFITM1^high^ cells have a reduced EV uptake ability. **A**, **B** Medium EV (mEV) uptake in IFITM1^low^ and IFITM1^high^ sorted cells. Representative images (left panels) and their quantification (right panels). Sorted cells were cultured 2D for 3 days and they were treated with mEVs derived from DiI-labelled HT29 CRC cells (**A**) or human colon fibroblasts (**B**). Note that mEVs show red fluorescence and phalloidin was used to visualize cells. Confocal microscope images were taken from two independent experiments. The shown images are optical slices from the inside of the cells. **C** Distribution of the DiI signal intensity in IFITM1^low^ and IFITM1^high^ cells. Cells were treated with mEVs derived from DiI-labelled fibroblasts. Note the shift of the curve between the two cell populations. **D** The fluorescent red signal of mEVs in IFITM1^low^ sorted cells, cultured for 3 days in 2D conditions. mEVs were collected from DiI-labelled fibroblasts and samples were treated with saponin or Triton X-100. Note that the EV signal disappears when using Triton X-100. **E** The sorting of CRC organoid cells pre-treated with DiI labelled fibroblast-derived small EVs (sEV) overnight in 2D cultures. **F** Relative RNA levels of *IFITM1, AXIN2* and *MYC* in the sorted EV+ and EV negative CRC cells (RT-qPCR, n = 3). RNA levels (normalized to housekeeping) of the EV+ samples were always taken as 1. **G** Relative RNA of the indicated genes in CRC organoid cells (cultured in 2D conditions) with or without fibroblast-derived sEVs (RT-qPCR, n = 3). **H** IFITM1 level and EV uptake of CRC organoid-derived cells (flow cytometry) in the presence/absence of fibroblast-derived mEVs. **I** The relative percentage of IFITM1^high^ and IFITM1^low^ cells within cells with mEV (left panel) or sEV (right panel) in four CRC organoid lines (flow cytometry). **J** EV-DiI red fluorescent signal intensity in IFITM1^−/low^ and IFITM1^high^ cells after treatment with mEVs or sEVs derived from fibroblasts (left panel) or HT29 cells (right panel). Four organoid lines were measured once or twice (flow cytometry). **K** IFITM1 level and the uptake of liposomes labelled with DiO (representative flow cytometry images). **L** DiO green fluorescent signal intensity within the DiO-liposome+ population (data were collected from four organoid lines in 4–5 experiments). Scale bars: 20 µm (**A**–**C**). Paired t-tests (**F**, **G**), t-test (**C**), Mann–Whitney U-test (**A**, **B**, **J**, **L**) were used with *p < 0.05, ***p < 0.005 and n.d.: p > 0.05

Interestingly, *Apc* mutant organoids that express a higher level of Ifitm1, accumulated less fibroblast-derived mEVs compared to wild-type intestinal organoids (Fig. S4f). To study whether EV uptake intensity differs between CRC cell subpopulations as well, we next sorted IFITM1^high^ and IFITM1^low^ cells and we cultured them with labelled mEVs or sEVs. Of note, less IFITM1^high^ cells took up EVs both in case of HT29 CRC cell-line-derived and human colon fibroblast-derived mEVs as compared to IFITM1^low^ CRC cells, detected by confocal microscopy (Fig. 5A, B). In addition, IFITM1^high^ cells that had taken up fibroblast-derived EVs showed a lower signal intensity for mEVs than IFITM^low^ cells (Fig. 5C). Thus, these results suggest that the difference in EV uptake is not restricted to one specific cell type-derived EVs. Importantly, we proved the presence of mEVs and sEVs in the conditioned medium of HT29 cells with NTA and transmission electron microscopy (Fig. S4g, h). In addition, when samples were treated with Triton X-100, the EV signal disappeared from the cells (Fig. 5D), proving that we visualized membrane-enclosed EVs in our experiments.

As the next experiment, we treated CRC organoid cells with fibroblast-derived labelled EVs in 2D cultures and then sorted cells with the highest and lowest fluorescent signal, representing cell populations with high and low EV uptake ability, respectively (Fig. 5E). Whereas we found no difference in the expression of *AXIN2* and *MYC*, we measured a significantly higher RNA level of *IFITM1* in cells with low EV uptake (Fig. 5F). Since we found no increase in the expression of *IFITM1* in CRC cells after treatment with EVs (Fig. 5G), this confirms again that IFITM1^high^ cells take up less EVs. In addition, the majority of cells with high IFITM1 levels were negative for the fluorescent signal after treatment with labelled fibroblast-derived mEVs (Fig. 5H). Furthermore, we observed the accumulation of IFITM1^low^ cells within the mEV+ cell population, measured by flow cytometry (Fig. 5I). When focusing on cells that had taken up mEVs, we detected a higher signal intensity, characterizing a higher mEV uptake, within the IFITM1^low^ cell population (Fig. 5J). Importantly, we obtained similar results when CRC organoid cells were treated with the sEV fraction collected from labelled fibroblasts or HT29 cells (Fig. 5I, J). Collectively, all these data prove (i) the presence of cell subclones in CRC with different mEV and sEV uptake ability and (ii) that IFITM1^high^ marks cells with a lower EV uptake.

Next we produced fluorescently labelled synthetic liposomes and we added them to sorted IFITM1^high^ and IFITM1^low^ CRC cells after ultracentrifugation. In contrast to the labelled or unlabelled liposome preparates, we detected no signal when only the fluorescent dye was ultracentrifuged (Fig. S4i), thus, ruling out the possibility that we used dye aggregates in our experiments. We found no statistically significant difference in the signal intensity between the two cell populations after adding fluorescently labelled synthetic liposomes (Fig. 5K, L). Thus, the difference in the uptake ability between the two CRC cell subpopulations is characteristic for the EVs.

### Fibroblast-derived EVs result in a marked increase in the proliferating cell number of IFITM1^low^ CRC organoids

**Fig. 6:**
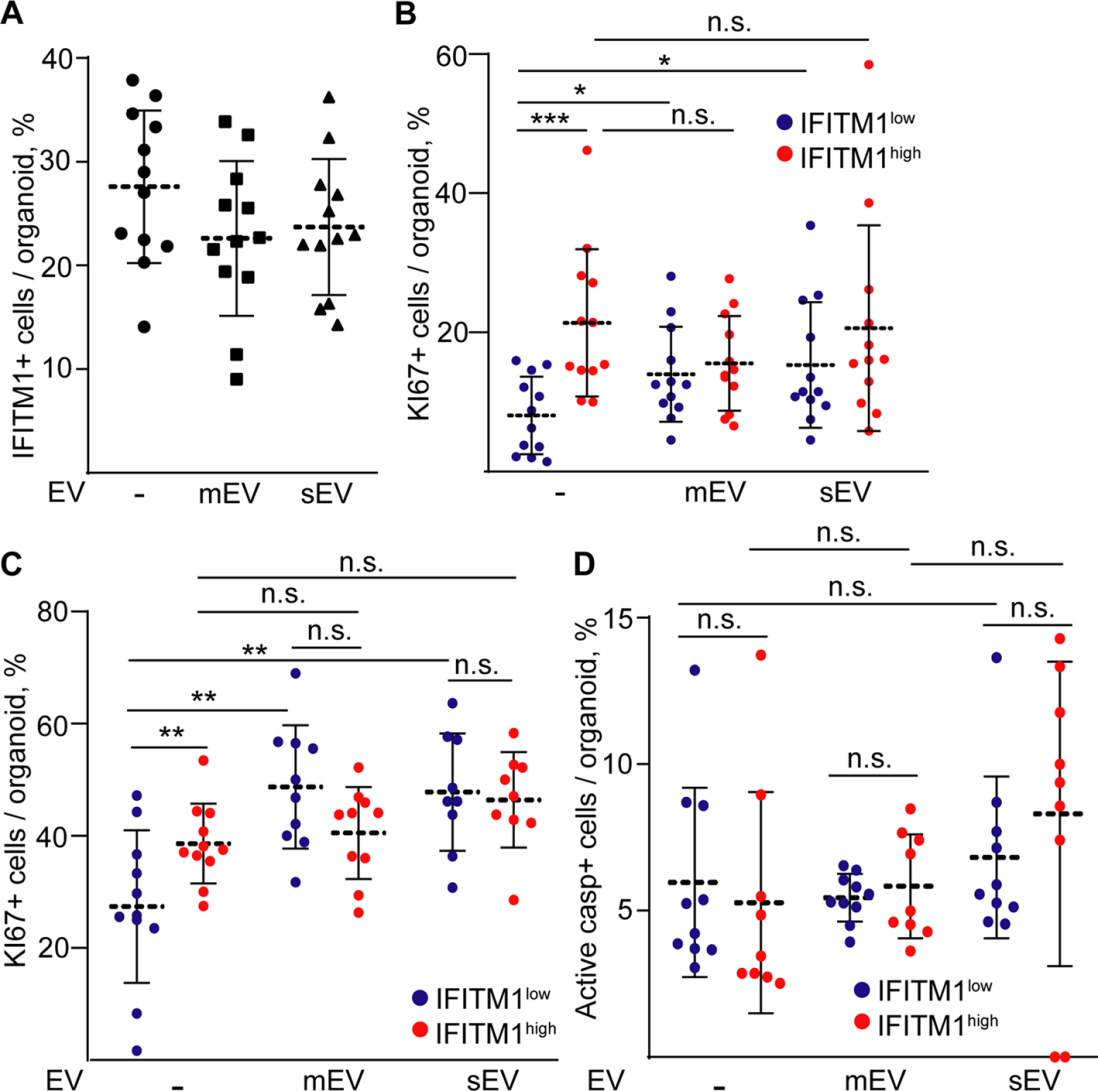
Fibroblast-derived EVs induce the proliferation of IFITM1^low^ CRC cells. **A** The proportion of IFITM1+ CRC cells in the absence of fibroblast-derived EVs or after adding sEVs or mEVs. **B** The percentage of IFITM1^low^/KI67+ and IFITM1^high^/KI67+ cells in CRC organoids after the indicated treatments. **C** The percentage of KI67+ proliferating cells in IFITM1^high^ or IFITM1^low^ CRC cell-derived organoids, treated with fibroblast sEVs or mEVs directly after sorting (whole-mount immunostaining and confocal microscope images). **D** The proportion of active caspase-3+ apoptotic cells in CRC organoids derived from the indicated cell populations, cultured with or without mEVs or sEVs. Images were taken from three experiments for **A**, **B**, **C** and **D**. Two-way ANOVA, and Tukey post hoc tests were carried out (**A**, **B**, **C**, **D**) with *p<0.05, **p < 0.01, ***p<0.005 and n.s.: p > 0.05. Note that only the relevant comparisons are shown

To test the functional relevance of differential EV uptake between CRC cell subpopulations, we first added fibroblast-derived mEVs or sEVs to CRC cells and we then detected IFITM1 expression and KI67 in the organoids after 7 days. Importantly, neither sEVs nor mEVs resulted in a change in the percentage of IFITM1+ CRC cells (Fig. 6A). However, we detected a markedly higher increase in the proportion of proliferating cells within the IFITM1- cell population than in IFITM1+ cells in the presence of EVs (Fig. 6B). Furthermore, adding fibroblast-derived mEVs or sEVs to sorted IFITM1^low^ cells resulted in organoids with a markedly higher increase in the number of KI67+ cells as compared to IFITM1^high^ cell-derived organoids (Fig. 6C). In addition, the initial difference in the percentage of proliferating cells between the two CRC cell subpopulations disappeared after treatment with mEVs or sEVs (Fig. 6C). On the other hand, we detected no changes in the percentage of active caspase-3+ apoptotic cells (Fig. 6D). Thus, these results indicate that differential mEV or sEV uptake by CRC cell subpopulations critically modifies the percentage of proliferating cells in an organoid model.

### Deleting IFITM1 results in a higher EV uptake in CRC organoid cells

**Fig. 7:**
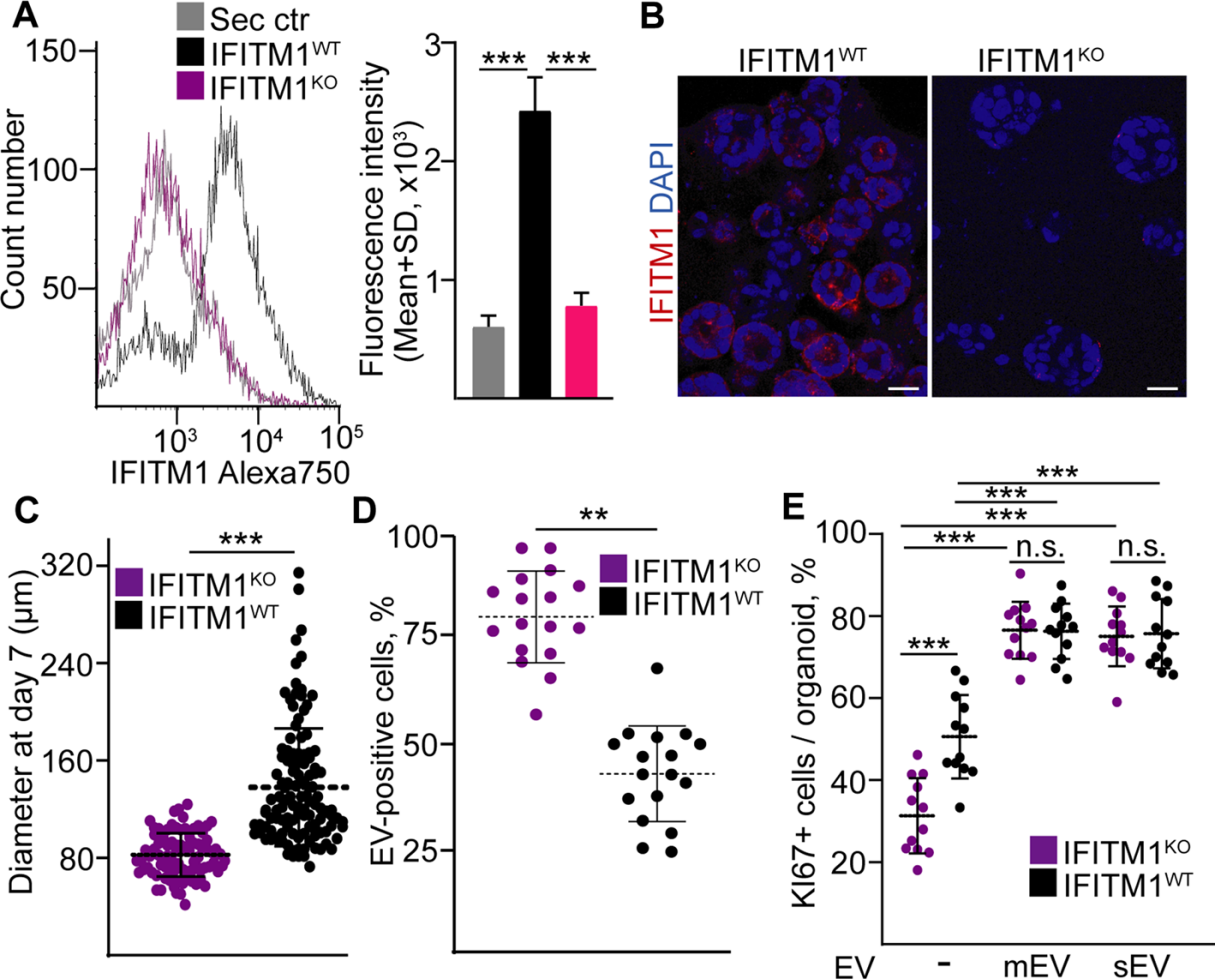
Inactivating IFITM1 results in a higher EV uptake. **A** Cell surface expression of IFITM1 on CRC organoid cells with (IFITM1^KO^) or without (IFITM1^WT^) CRISPR-based inactivation of the molecule (flow cytometry, Ctr: isotype control from IFITM1^WT^ cells). Representative histograms (left panel) and their quantification are shown (right panel, n = 4, GeoMean values were analyzed). **B** Immunostaining of CRC organoids derived from IFITM1^WT^ or IFITM1^KO^ cells (confocal microscopic images). **C** The diameters of IFITM1^WT^ and IFITM1^KO^ organoids on day 7 of 3D culturing. **D** The percentage of CRC cells with labelled EV signal in 2D cultures. Cells were isolated from organoids with the indicated genetic background and they were treated with labelled fibroblast-derived mEVs (evaluation of confocal images from four organoid lines). **E** The percentage of KI67+ organoid cells in the absence of EVs and in the presence of fibroblast-derived mEVs or sEVs (quantification of confocal microscopic images from four organoid lines). Unpaired t-test (**A**), Mann–Whitney U-test (**C**,**D**) or Kruskal–Wallis and Dunn tests (**E**) were used. Scale bars: 50 µm (**B**). **p<0.01, ***p<0.005, and n.s.: p>0.05

To test whether IFITM1 is functionally important for the enhanced proliferation rate and reduced EV uptake of cells with IFITM1 expression, we inactivated this gene in CRC organoids. Flow cytometry and immunocytochemistry proved the largely diminished level of IFITM1 in these cells (Fig. 7A, B). Of note, we observed a decreased diameter of organoids established from IFITM1^KO^ cells (Fig. 7C). When using labelled human colon fibroblast EVs, we detected a markedly higher percentage of EV-positive cells derived from IFITM1^KO^ organoids compared to IFITM1^WT^ cells (Fig. 7D), indicating that the lack of IFITM1 induced EV uptake. Similarly to the IFITM1^low^ and IFITM1^high^ populations, IFITM1^KO^ organoids showed a reduced percentage of KI67+ cells compared to IFITM1^WT^ organoids (Fig. 7E). However, fibroblast-derived mEVs or sEVs had a larger effect on the proliferation of IFITM1^KO^ organoids (Fig. 7E), indicating the functional effect of the increased EV uptake of cells without IFITM1. Collectively, these data suggest that IFITM1 is not only a marker of a CRC cell population with reduced EV uptake ability, but this molecule is also functionally involved in this process.

## Discussion

EVs can be edited and loaded with specific molecules. Although they are considered as a promising tool for cancer therapy, it is still unknown which CRC cell populations take up EVs preferentially. By using patient-derived organoids, here we identify CRC subpopulations with different EV uptake capabilities. Importantly, whereas CRC cells expressing the cell surface protein IFITM1 at a high level (IFITM1^high^) and at a low level (IFITM1^low^) do not differ in their EV release intensity, IFITM1^high^ cells take up significantly less EVs. Of note, deleting IFITM1 leads to the marked increase in EV uptake, proving that IFITM1 is not only a marker of a CRC cell population, but is also actively involved in this process. Fibroblast-derived EVs result in a marked increase in the proliferating cell number of IFITM1^low^ CRC organoids, showing the functional significance of the difference in EV uptake. We also show that *Ifitm1* expression is increased after *Apc* mutation, similarly to human adenomas when compared to normal colonic epithelium. CRC patient-derived organoids have a heterogeneous intra-organoid expression of IFITM1 and the IFITM1^high^ CRC population contains more proliferating cells.

IFITM family members have been identified as molecular markers in human colorectal tumors [29]. Other published studies have also connected IFITM1 to the progression of colorectal cancer, showing that it modifies the proliferating, invasive, and metastatic capability of CRC cell lines [30]. However, the majority of these previous data were derived from models using cell lines cultured 2D. Importantly, our study is the first where patient-derived organoids were used to characterize IFITM1^+^ CRC cells. We observed that IFITM1^high^ sorted cells form organoids with more proliferating cells, suggesting that IFITM1^high^ and IFITM1^low^ subpopulations differentially contribute to CRC tumorigenesis. Interestingly, we found no difference in the RNA level of some CRC stem cell and Wnt target genes, indicating that IFITM1 may regulate proliferation not directly via the Wnt pathway. In line with these data, loss of IFITM1 markedly increased nuclear p21 level, leading to reduced proliferation in breast cancer [31]. In another study, Provance et al. found that targeting IFITM1 inhibited the proliferation of breast cancer cells via NFκB [32].

*IFITM* genes are generally regulated by type I and type II interferons. Interestingly, however, IFITM3 is constitutively expressed in tissue‐resident T cells in some tissues, such as the airways and the lungs [33]. In T cells, IFITM2 and IFITM3 expressions are regulated by T‐cell receptor (TCR) signal transduction [34], and hedgehog (Hh) signalling modifies the expression of *IFITM* genes in some systems [35]. This shows diverse regulatory mechanisms for this gene cluster. Here we prove that human organoids derived from adenomas or CRC patients express *IFITM1* at a higher level compared to the normal colon. By using genetically modified mouse intestinal organoids, we provide evidence that *Apc* mutation is a critical factor in the regulation of *Ifitm1* expression. Our findings concur with those of Lickert et al. [36] who found a regulatory effect of the Wnt pathway on *Ifitm* genes during gastrulation. In addition, we confirmed the results by Andreu et al. [37], showing the regulation of many *Ifitm* members in mouse intestinal adenoma models. Interestingly, however, we found no difference in IFITM1 expression in the LGR5^high^ CRC cells compared to LGR5^low^ cells. Although LGR5^high^ cells represent a CRC cell population with stem cell features and high Wnt activity [5], this finding suggests that IFITM1 expression is regulated not only by the strength of the Wnt activation, but other signalling pathways may have a critical influence as well.

The *IFITM* genes encode for a group of small homologous proteins that are localized in the membranes of the endosomal-lysosomal compartment or the plasma membrane [38]. While there are seven *Ifitm* genes in mice, only five of them have been identified in the human genome. However, *IFITM1* is among the genes shared by both species. IFITM proteins provide a cellular resistance against a wide variety of both enveloped and non‐enveloped viruses not only in vitro, but in vivo as well [39, 40]. Similarly to certain viruses, EVs contain a membrane envelope. Although the mechanism of action of the IFITM proteins is largely unknown, some studies suggested that they exert their anti-viral effects via changing the fluidity or other physical properties of biological membranes [41, 42] which may apply for EV uptake as well. Of note, here we show that IFITM1^high^ cells take up less EVs compared to cells with low IFITM1 expression. Thus, our results indicate the existence of differential EV uptake within CRC. Importantly, by inactivating IFITM1, we also demonstrate that this molecule is functionally important for this process. Thus, these data provide further evidence for the relationship between certain viruses and EVs not only in the mechanism of their production, but in their uptake as well [43]. Of note, the IFITM1^low^ population contained less proliferative cells compared to the IFITM1^high^ population. However fibroblast-derived EVs diminished this difference between IFITM1^high^ and IFITM1^low^ cells. They had a more pronounced stimulatory effect on cell proliferation in the IFITM1^low^ CRC subpopulation that accumulated more EVs. These data not only highlight the complex regulation of proliferation in CRC, but also illustrate the biological importance of the differential EV uptake by tumor cells.

EVs contain proteins in their membranes that enhance their uptake, thus, providing a powerful delivery system of drugs or RNAs in RNA interference (RNAi)-based approaches. The usability of this approach has recently been proven by two elegant studies where RNAi was delivered to specifically target oncogenic KRas in pancreatic tumors in mouse models [44, 45]. Importantly, these results open the possibility for engineered EV-based therapies targeting oncogenes in other cancers, such as CRC. However, the proper and efficient use of EVs delivering the engineered cargo critically depends on their uptake by tumor cells. Importantly, the heterogeneous level of IFITM1 on CRC cells may result in the blockade of EV-mediated drug delivery in some CRC cells. Furthermore, the *Apc* mutation-induced *Ifitm1* expression and the higher level of IFITM1 in CRC cells compared to normal colon epithelial cells raise the possibility that normal colon epithelial cells may be targeted even more efficiently by EVs.

**Fig. 8:**
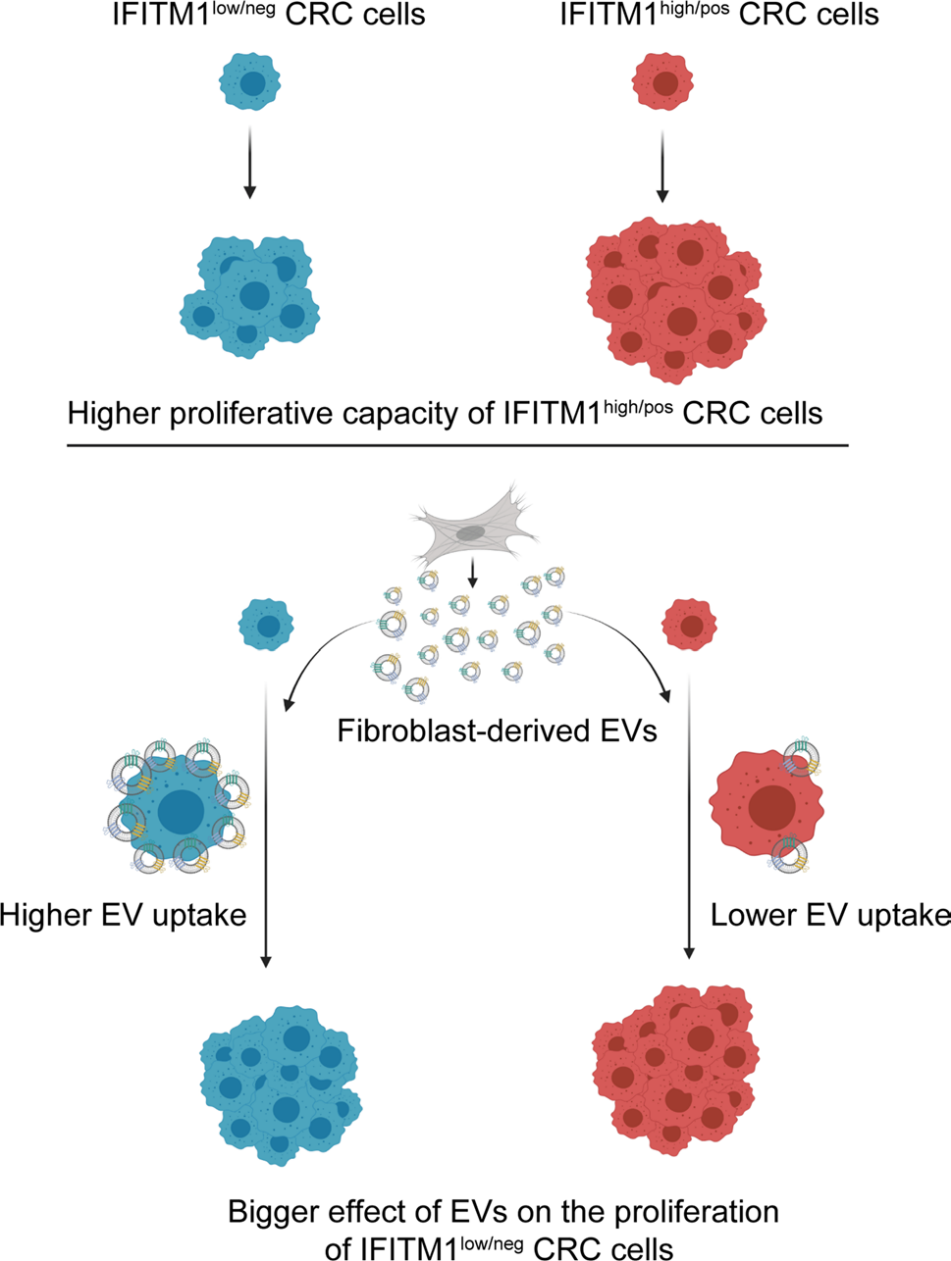
Differential EV uptake by IFITM1^low^ and IFITM1^high^ CRC cells has an important effect on the proliferation intensity

Collectively, we provide evidence that IFITM1 inhibits EV uptake in CRC cells. Although IFITM1^low/neg^ cells have a lower proliferation potential, the addition of EVs has a bigger effect on their division compared to IFITM1^high^ cells (Fig. 8). Thus, IFITM1-regulated EV uptake intensity is a critical factor for modifying important tumorigenic features of some CRC cells, such as proliferation. Since this fact may influence the effect of EVs in clinical applications, it must be considered when designing EVs as therapeutic tools in CRC.

## Supplementary Information

Below is the link to the electronic supplementary material.

Supplementary file 1 (PDF 5246 KB)

## Data Availability

Data are available from the corresponding author upon reasonable request.
