# Supplementary material for: IFITM1 expression determines extracellular vesicle uptake in colorectal cancer
Source: Cell Mol Life Sci. 2021 Oct 5;78(21-22):7009–24. doi: 10.1007/s00018-021-03949-w (PMC8558170; doi:10.1007/s00018-021-03949-w)
Supplement: Supplementary file 1 — (PDF 5246 KB) [file 18_2021_3949_MOESM1_ESM.pdf]

IFITM1 expression determines extracellular vesicle uptake in colorectal cancer

Andrea Kelemen, Idan Carmi, Ádám Oszvald, Péter Lőrincz, Gábor Petővári, Tamás Tölgyes, Kristóf Dede, Attila Bursics, Edit I. Buzás, Zoltán Wiener*

*corresponding author

No	Normal colon sample	Adenoma sample	CRC sample	Age	Sex	Diagnosis	TNM	Grade
1	-	-	+	63	F	adenocarcinoma	T3N0M0	2
2	-	-	+	74	M	adenocarcinoma	T3N0M0	2
3	-	-	+	75	M	adenocarcinoma	T3N2aM0	2
4	-	-	+	64	M	adenocarcinoma	T3N1bM0	1
5	+	-	-	56	M	adenocarcinoma	yT2N0	1
6	+	-	-	76	F	adenocarcinoma	T2N1a	1
7	+	+	-	67	F	colon adenoma	----	-
8	-	+	-	55	F	colon adenoma	----	-

Table S1. Patient data for CRC, adenoma and normal colon organoid cultures. CRC organoids #1-3 have been published and extensively characterized in our previous studies [1]. Note that sample #4 required EGF (showing the presence of normal KRAS pathway) and it was sensitive to nutlin-3 treatment, indicating the absence of two mutant *TP53* alleles [2]. Sequencing of the DNA binding domain of *TP53* did not identify mutations.

Antibody	Source	Clone/Cat No
FITC anti-human CD81	Molecular Probes	A15753
PE anti-human CD63	Sigma	SAB4700218
anti-KI67	Abcam	Ab16667
anti-human/mouse active caspase-3	R&D Systems	AF835
anti-human IFITM1 C-terminal	Abcam	ab117519
anti-human IFITM1 N-terminal	Sigma-Merck	HPA004810
anti-mouse IgG Alexa 488	Invitrogen/Thermo Fisher	AF21202
anti-mouse IgG Alexa 568	Invitrogen/Thermo Fisher	AF10037
anti-rabbit IgG Alexa 488	Invitrogen/Thermo Fisher	A21206
anti-rabbit IgG Alexa 568	Invitrogen/Thermo Fisher	A11011
anti-rabbit IgG Alexa 750	Invitrogen/Thermo Fisher	A21039
anti-goat IgG Alexa 488	Invitrogen/Thermo Fisher	A21467
anti-goat IgG Alexa 568	Invitrogen/Thermo Fisher	A11057S

Table S2. Antibodies used in our studies.

**Primer name**	**Sequence**
hLGR5_fw	AGTGCTGTGCATTTGGAGTG
hLGR5_rev	AGGGCTTTCAGGTCTTCCTC
hAXIN2_fw	CTGGCTATGTCTTTGCACCA
hAXIN2_rev	CTTCACACTGCGATGCATTT
hMYC_fw	TCCTCGGATTCTCTGCTCTC
hMYC_rev	CTCTGACCTTTTGCCAGGAG
hIFITM1_fw	CTTGAACTGGTGCTGTCTGG
hIFITM1_rev	AATCAGGGCCCAGATGTTCA
hVIM_fw	GGTACTCGCATTCTCCACCT
hVIM_rev	CTCAATGTCAAGGGCCATCT
hZEB1_fw	GCTGACTGTGAAGGTGTACC
hZEB1_rev	ACATCCTGCTTCATCTGCCT
hCD133_fw	GCCTCTGGTGGGGTATTTCT
hCD133_rev	TACCTGGTGATTTGCCACAA
hMUC2_fw	ATCCTCAAAAGCAGCGTGTT
hMUC2_rev	CCCCCTCTTTGGTACACTCC
hALPI_fw	TCCCATGTCTTCTCCTTTGG
hALPI_rev	CTCGCTCTCATTCACGTCTG
hHPRT1_fw	TGAGGATTTGGAAAGGGTGT
hHPRT1_rev	TCCCCTGTTGACTGGTCATT
mLgr5_fw	CCTGTCCAGGCTTTCAGAAG
mLgr5_rev	CTGTGGAGTCCATCAAAGCA
mAxin2_fw	CTCCCCACCTTGAATGAAGA
mAxin2_rev	ACTGGGTCGCTTCTCTTGAA
mMyc_fw	TCCTGTACCTCGTCCGATTC
mMyc_rev	GGTTTGCCTCTTCTCCACAG
mProx1_fw	GCTATACCGAGCCCTCAACA
mProx1_rev	ATCCAGCTTGCAGATGACCT
mIfitm1_fw	AGCCTATGCCTACTCCGTGA
mIfitm1_rev	AATGGCACAGACAACGATGA
mHprt_fw	GCGATGATGAACCAGGTTATGA
mHprt_rev	GCCTCCCATCTCCTTCATGA

Table S3. Primers used for RT-qPCR.

Supplementary references

1. Szvicsek Z, Oszvald A, Szabo L, Sandor GO, Kelemen A, Soos AA, Paloczi K et al (2019) Extracellular vesicle release from intestinal organoids is modulated by Apc mutation and other colorectal cancer progression factors. Cell Mol Life Sci 76:2463-2476.
2. Drost J, van Jaarsveld RH, Ponsioen B, Zimberlin C, van Boxtel R, Buijs A, Sachs N et al (2015) Sequential cancer mutations in cultured human intestinal stem cells. Nature 521:43-7.

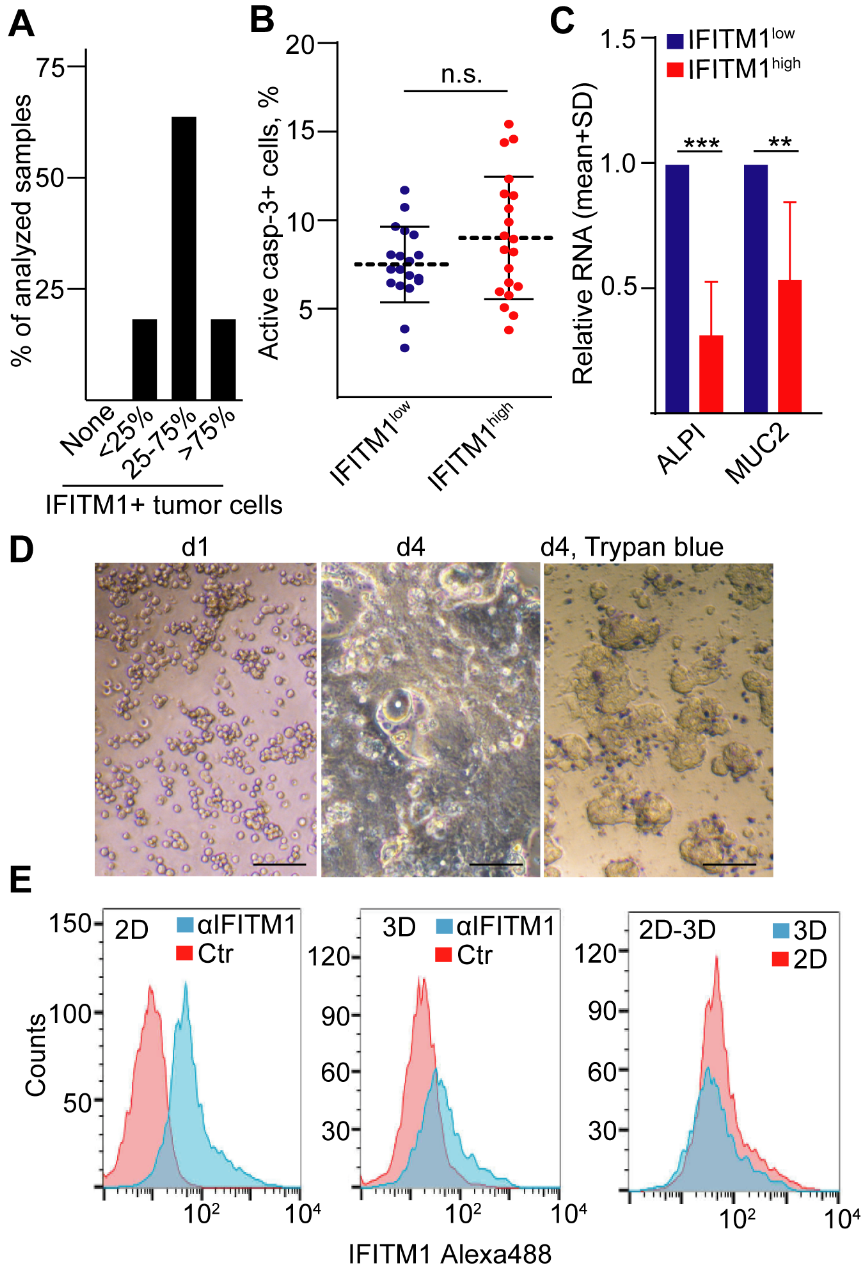

Figure S1. IFITM1^{high} CRC cell-derived organoids have a lower RNA level for intestinal differentiation markers. A) The percentage of samples showing the indicated proportion of IFITM1 CRC cells (analysis of data from Protein Atlas, www.proteinatlas.org). B) The percentage of active caspase-3+ apoptotic cells in organoids derived from IFITM1^{high} or IFITM1^{low} CRC cells (whole-mount immunostaining). Images from three experiments were analyzed. C) The relative RNA of ALPI (enterocyte marker) and MUC2 (Goblet cell marker) in organoids produced from sorted cells (RT-qPCR, n=3). D) Images from dissociated CRC organoid cells cultured in 2D conditions for one and four days and their viability on day 4 (Trypan blue dye exclusion test). E) IFITM1 surface level on CRC cells cultured 2D or 3D in Matrigel for 4 days (flow cytometry of unfixed cells, Ctr: isotype antibody control). Scale bars: 50 µm (D). Mann-Whitney U-test (B) or paired t-test (C) were used with $^{**}p<0.01$, $^{***}p<0.005$, n.s.: $p>0.05$.

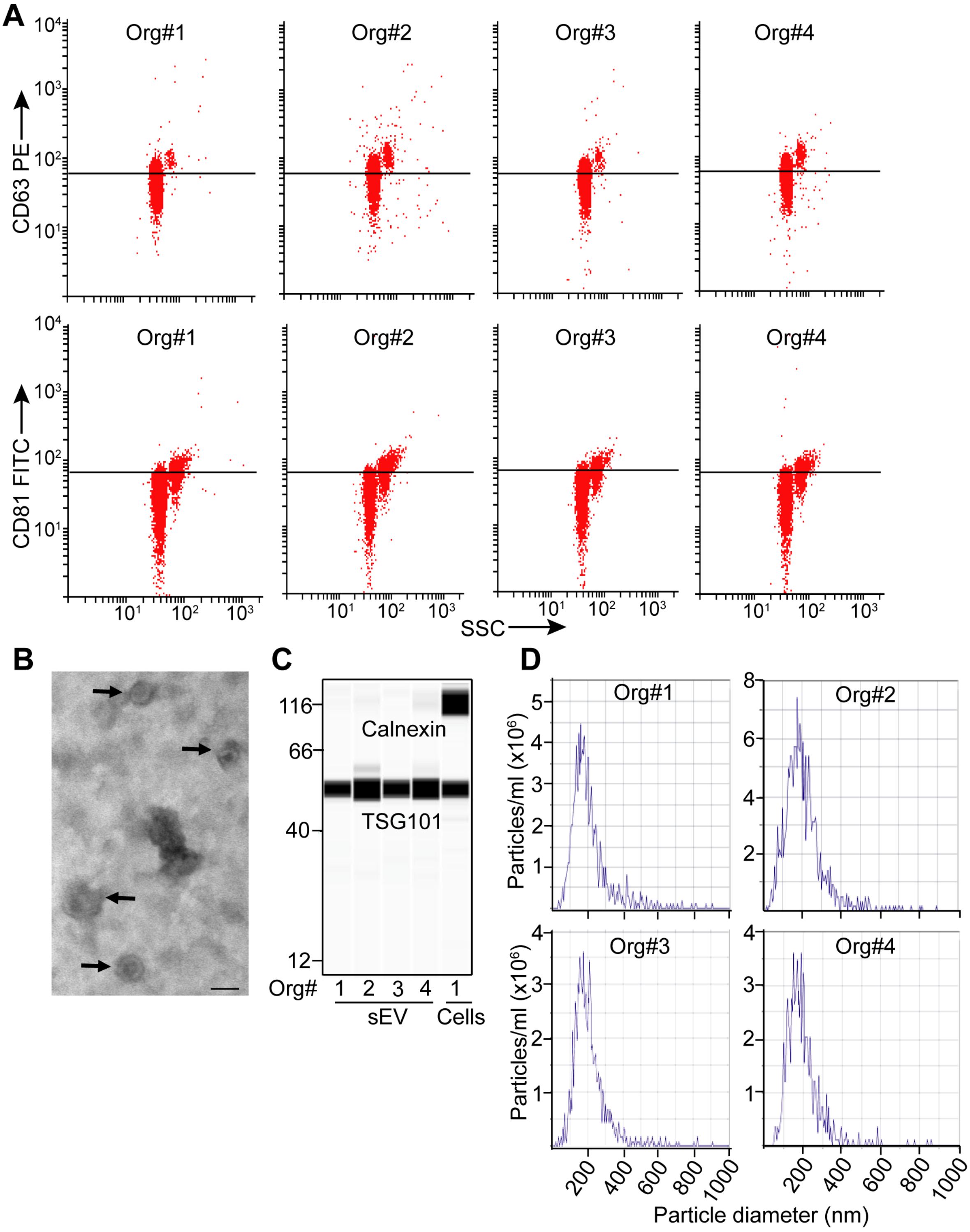

Figure S2. Characterizing the CRC organoid-derived EVs. A) Flow cytometry of anti-CD63 or anti-CD81-coated beads incubated in organoid-derived conditioned medium. Beads with EVs were detected with anti-CD63 or anti-CD81. B) Transmission electron microscopic image from an ultracentrifuged organoid conditioned medium. The arrows mark small EVs (sEV). C) Capillary-based immunoblot analysis of ultracentrifuged supernatants from four organoid lines and from the cell lysate of an organoid culture. Note that in contrast to the sEV marker TSG101, calnexin is not present in the sEV preparates. D) Representative NTA measurements from the ultracentrifuged supernatants (containing sEVs) of organoid cultures. Scale bar: 100 nm (B).

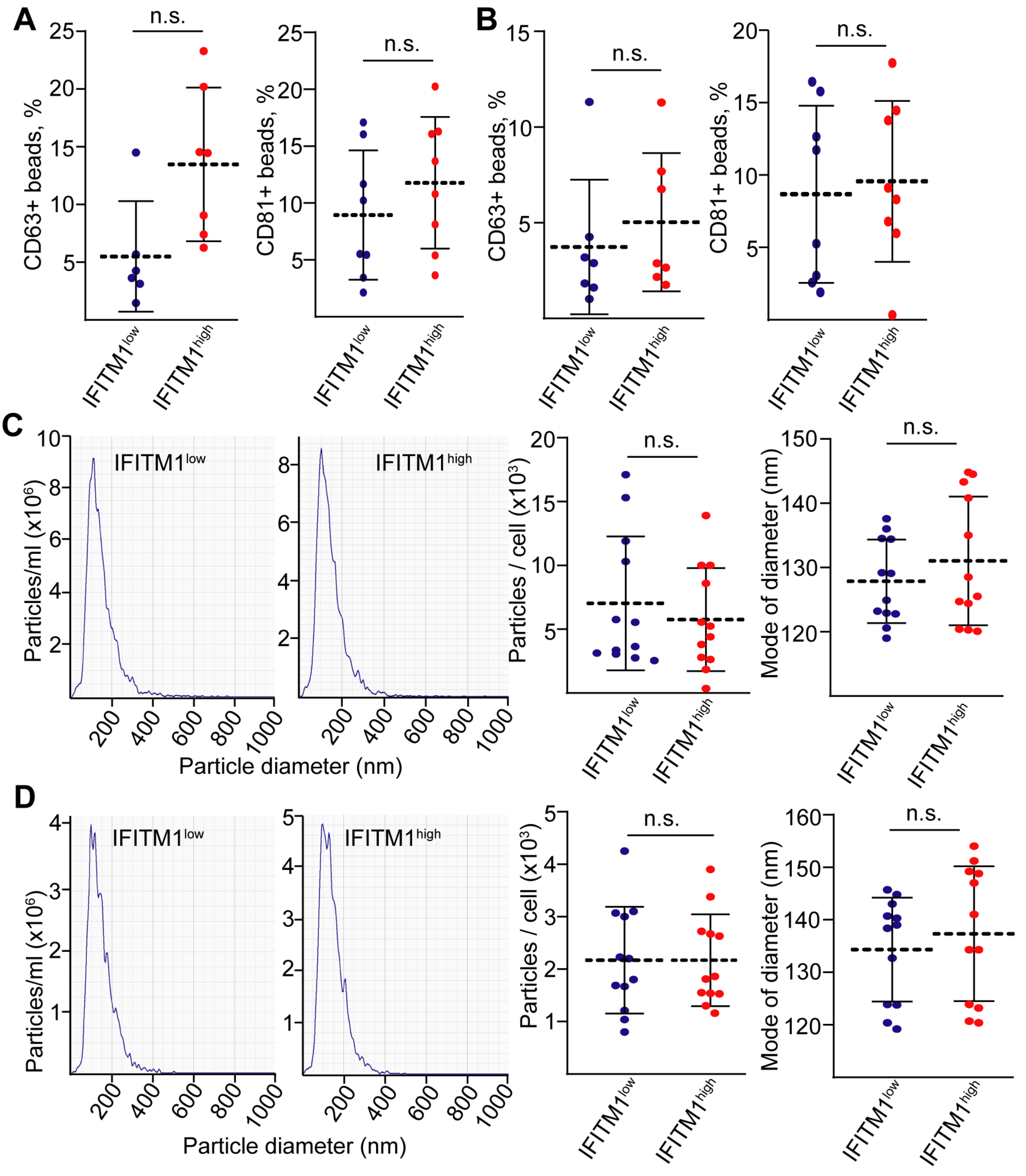

Figure S3. There is no difference in the EV release between IFITM^{high} and IFITM^{low} CRC cell-derived organoids in collagen or in Matrigel/collagen. A-B) The percentage of positive anti-CD63 or anti-CD81-coated beads after incubation in organoid-derived supernatants (flow cytometry). Organoids were cultured in 50% Matrigel/50% collagen I (A) or in collagen I (B) and beads with EVs were detected with anti-CD63 or anti-CD81. C-D) Representative NTA measurements and the quantification of particle numbers (normalized to cell number) and their size (mode). IFITM1^{high} and IFITM1^{low} cell-derived organoids were cultured in Matrigel/collagen I (C) or in collagen I (D), the conditioned medium was centrifuged at 12,500g for 20 min and the supernatants containing sEVs were applied to NTA measurements. Mann-Whitney U-test (A, B, C, D) was used with n.s.: $p>0.05$.

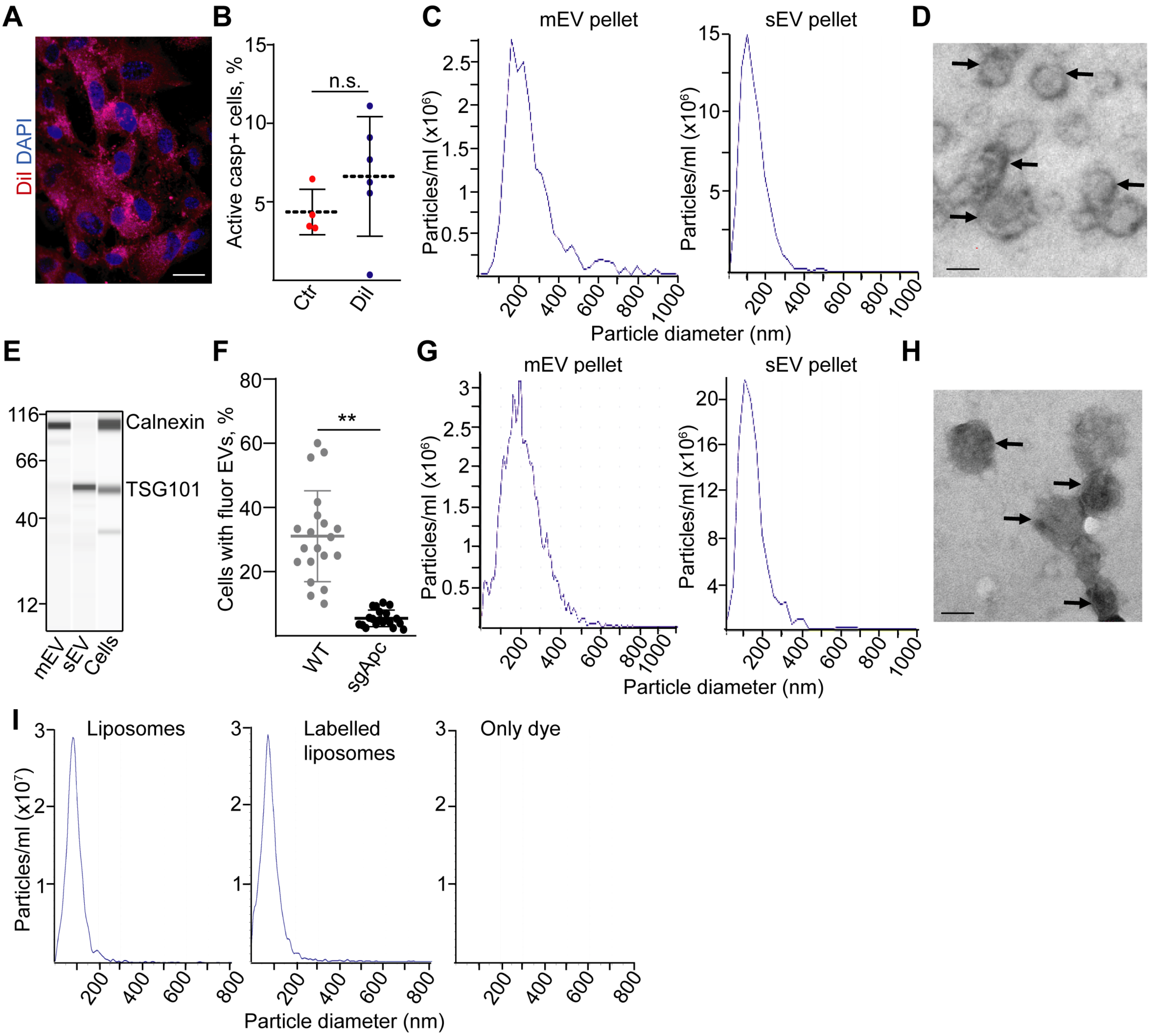

Figure S4. Apc mutant intestinal organoid cells take up less EVs compared to WT cells. A) Colon fibroblasts after labelling with DiI (confocal microscope). B) The percentage of active caspase+ apoptotic fibroblasts without (Ctr) and with DiI labelling (immunostaining and confocal microscopy, images were taken from parallel experiments). C) NTA measurements from human colon fibroblast-derived 12,500g pellet (left panel, mEV) and from the pellet after ultracentrifugation (right panel, sEV). D) Transmission electron microscopy (TEM) from an ultracentrifuged fibroblast culture supernatant. The arrows mark sEVs. E) Capillary-based Western-blotting analysis of centrifuged (mEV), ultracentrifuged (sEV) fibroblast EV samples and a cell lysate for the indicated proteins. F) The percentage of wild type (WT) and Apc mutant (sgApc) mouse intestinal organoid cells with red fluorescent EV signal (confocal images from three experiements were evaluated). Note that mEVs were isolated from fibroblasts that had been pre-treated with the red fluorescent membrane labelling dye DiI. G) NTA measurements from the centrifuged mEV and ultracentrifuged sEV fractions of HT29 CRC cell culture suernatants. H) TEM image from the pellet after ultracentrifuging the conditioned medium of HT29 cell cultures. The arrows mark EVs. I) NTA measurements from the UC pellet derived from liposomes, labelled liposomes and DiO dye solution without liposomes. Scale bars: 50 µm (A), 100 nm (D, H). Mann-Whitney U-test was used (B, F) with $^{**}p<0.01$ and n.s>0.05.
